# Oral Candidiasis: Aiding in the Diagnosis of HIV—A Case Report

**DOI:** 10.1155/2011/929616

**Published:** 2011-09-15

**Authors:** Arvind Shetti, Ishita Gupta, Shivyogi M. Charantimath

**Affiliations:** Department of Oral Medicine & Radiology, KLE VK Institute of Dental Sciences, Nehru Nagar, Belgaum 590010, India

## Abstract

Opportunistic fungal infections account for a significant amount of morbidity associated with HIV disease. Candidiasis is the most common oral opportunistic infection affecting people with HIV infection or AIDS. It is considered as an important marker of immune suppression and may be the initial manifestation of the disease in about 10% of HIV-infected adults. We report a case of an apparently healthy 45-year-old male with oral candidiasis which proved to be the first indicator of HIV infection.

## 1. Introduction

Acquired immune deficiency syndrome (AIDS), a disease of the human immune system caused by the human immunodeficiency virus (HIV), has emerged as a global crisis since its discovery in the summer of 1981 in the United States. Defective cellular immunity associated with AIDS may place the infected person at risk for a variety of opportunistic infections. Oral candidiasis is one of the most common, treatable oral mucosal infections seen in persons with HIV or AIDS. 

The infection is caused by *Candida albicans*, a dimorphic fungal organism that is typically present in the oral cavity in a nonpathogenic state in about one-half of healthy individuals but under favorable conditions, has the ability to transform into a pathogenic (disease causing) hyphal form. Conditions that favor this transformation include broad-spectrum antibiotic therapy, corticosteroids, xerostomia, immune dysfunction, diabetes mellitus, nutritional deficiencies, or the presence of removable prostheses [1]. Here we present a case of a 45 year old male who presented with oral candidiasis which led to the diagnosis of HIV infection.

## 2. Case Report

A 45-year-old male reported to the Department of Oral Medicine and Radiology with a chief complaint of burning sensation on the tongue and cheeks from the past 3 months. He had no significant past medical and drug history. The patient gave a history of smoking 5 cigarettes/day for 15 years but had quit the habit 6 months back. 

**Figure 1:**
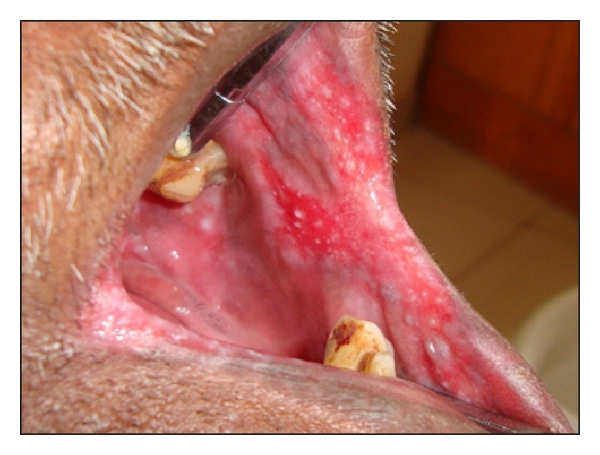
Intraoral picture showing the left retrocommissural area and buccal mucosa.

**Figure 2:**
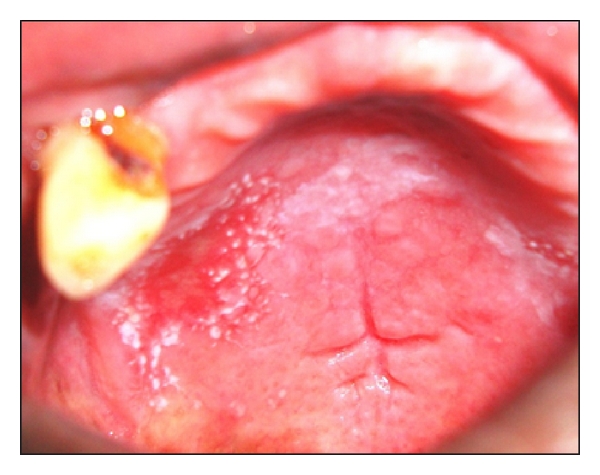
Intraoral picture showing the palate.

**Figure 3:**
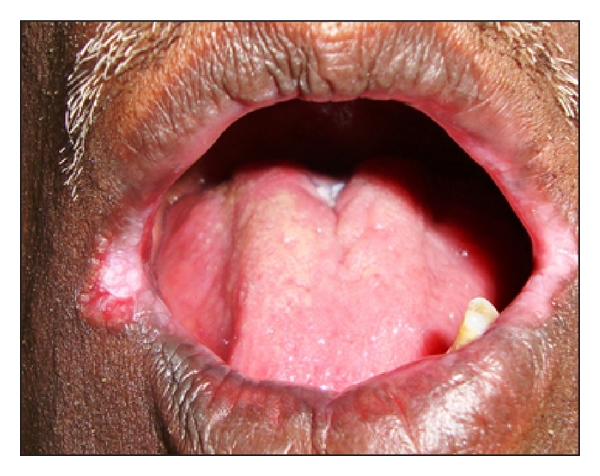
Angular cheilitis on the right and left commissures.

Intraoral examination revealed erythematous patches on the right and left retrocommissural areas (Figure 1) extending 2 cms posteriorly into the buccal mucosa and 2 cms superiorly and inferiorly. The erythematous area was superimposed with nodular white projections that were nonscrapable. Similar patch was present on the palate (Figure 2). A nonscrapable hyperkeratotic patch measuring 1 × 1 cm was also present on the dorsum of the tongue, and angular cheilitis was present bilaterally on the lip commissures (Figure 3). Multiple teeth were found to be missing. The remaining teeth had poor periodontal status. 

**Figure 4:**
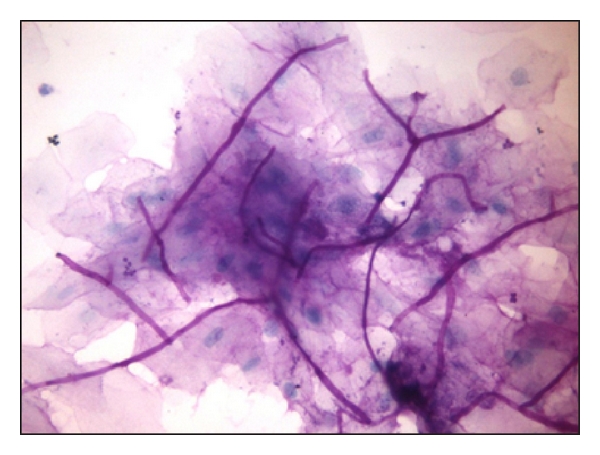
Photomicrograph of the exfoliative smear (40x) showing candidal hyphae.

The above clinical features and history led to a provisional diagnosis of erythematous candidiasis. An exfoliative smear was then prepared utilizing periodic acid schiff stain which revealed many epithelial cells with candida-like hyphae and spores confirming the diagnosis of candidiasis (Figure 4). Subsequently the patient was prescribed topical antifungal (clotrimazole) and topical anesthetic (benzydamine hydrochloride). The lesions on the right and left buccal mucosa showed improvement within 14 days; however, no improvement was seen on the palate and tongue. When the patient failed to respond to treatment, an underlying immunodeficiency was suspected. On being questioned about his lifestyle, the patient reluctantly admitted having unprotected sex with multiple partners. This prompted an HIV ELISA test which returned as positive. Confirmatory tests performed for HIV were positive, and CD4 count was 272 cells/mm^3^. Thus, oral candidiasis revealed the underlying HIV infection following which the patient was managed with appropriate systemic antifungals (ketoconazole) along with topical antifungals (clotrimazole) and appropriate antiretroviral therapy. 

## 3. Discussion

HIV infection is characterized by progressive immunosuppression due to low absolute CD4 counts and the perturbed cytokine network which manifest havoc at clinical level. The clinical consequences of HIV infection encompass a spectrum ranging from an acute syndrome associated with primary infection to prolonged asymptomatic state to advanced disease (Table 1). The oral health status of an HIV-infected patient at presentation is an extremely important parameter, as it may reveal important information regarding the immune status of the individual. Oral disorders occur in about 64–80% cases of HIV/AIDS in India [2] and may present as a wide range of lesions, notably fungal, viral, and bacterial infections and malignant neoplasms such as Kaposi's sarcoma and nonspecific presentations such as aphthous ulcerations and salivary gland disease as would be expected in severe defect of T-lymphocyte-mediated immunity. Factors which predispose expression of oral lesions include CD4 counts less than 200 cells/mm^3^, viral load greater than 3000 copies/mL, xerostomia, poor oral hygiene, and smoking [3].

The most common HIV-related oral disorder is oral candidiasis which occurs in 17–43% cases with HIV infection and in more than 90% of cases with AIDS [4]. Oropharyngeal candidiasis is among the initial manifestations of HIV-induced immunodeficiency and typically affects the majority of persons with advanced untreated HIV infection. Presenting months or years before more severe opportunistic infections, it may be a sentinel event indicating the presence or progression of HIV disease.

Infection with *Candida albicans* presents mainly four forms: pseudomembranous candidiasis, hyperplastic candidiasis, erythematous candidiasis, and angular cheilitis. Patients may exhibit one or a combination of any of these presentations. In patients with fully blown AIDS, the pseudomembranous form of candidiasis is most common, while in patients infected with HIV, the erythematous type is dominant [3, 5, 6] as was seen in the present case. Erythematous candidiasis presents as red macular lesions typically on the palate and dorsum of the tongue. Pseudomembranous candidiasis appears as creamy white curd-like plaques on the buccal mucosa, tongue, and other oral mucosal surfaces that can be wiped away, leaving a red or bleeding underlying surface while the hyperplastic type of oral candidiasis is characterized by white plaques that cannot be removed by scraping and is common in the buccal mucosa. Angular cheilitis presents as cracking, peeling, or ulceration involving the corners of the mouth and is frequently present in combination with other forms of candidiasis. 

HIV infection presents with a plethora of oral manifestations which are shown by all patients at some point of their disease. It has been shown by various studies on HIV and AIDS that oral candidiasis is the most common opportunistic infection [2, 4]. These oral manifestations can also be the initial indicator of underlying HIV infection. In our case, the patient appeared apparently healthy and was completely unaware of his immunologic status. It was the burning sensation on the tongue and cheeks which made him obtain a dental opinion. The patient presented with the typical features of erythematous candidiasis including burning sensation along with angular cheilitis, and these findings triggered investigations for HIV infection. This discovery was similar to the cases observed in the past where candidiasis was the sole initial manifestation of HIV infection leading to its diagnosis [7, 8]. There also have been reports where the rarer oral infection of histoplasmosis has aided in identifying the HIV status of an individual [9, 10]. Tuberculosis was found to be the most frequently occurring systemic coinfection in AIDS [6].

Identification of the fungal pseudohyphae within exfoliative cytologic preparations, often utilizing periodic acid schiff and/or-Papanicolaou-stained preparations, is the optimal standard for the diagnosis of all candidiasis, although the highest yield of positive cytology smears is with pseudomembranous candidiasis [11]. In general, the frequency of isolation of candida species increases with increasing severity of HIV disease and with lower CD4 : CD8 ratio [12]. Oral manifestations especially candidiasis has been found to be significantly correlated to a reduced CD4 cell count below 200 cells/mm^3^ [3, 6]. Management is based on the extent of the infection with topical therapies consisting of clotrimazole troches, nystatin oral suspension, and nystatin pastilles utilized for mild to moderate cases. Systemic agents are reserved for moderate to severe disease and include fluconazole, the most widely used drug, itraconazole, and voriconazole; the latter should be reserved for fluconazole-resistant cases. HIV-infected patients usually have associated esophageal candidiasis along with oral candidiasis and hence require a longer and higher dose of antifungals [12]. Undeniably, it was the presence of erythematous candidiasis, angular cheilitis, and periodontitis and the unresponsiveness of the patient to topical antifungals that prompted us to elicit his lifestyle habits and carry out investigations leading to a diagnosis of HIV infection.

## 4. Conclusion

Oral lesions serve as early marker for HIV infection and may herald deterioration in general health and a poor prognosis. The dentist must be well aware of the characteristics and presentation of the manifestations of HIV infection, thus enabling early identification of HIV, ensuring timely initiation of therapy. A candidal infection may often be the first clinical sign of HIV infection. The presence of oral candidiasis without a local cause, such as xerostomia or therapy with antimicrobials, corticosteroids, or other immune suppressive drugs in a person who otherwise appears healthy should prompt investigation into lifestyle and other factors pertaining to the risk of HIV infection. The oral manifestations thus can be used as a marker of immune status for field-based settings in developing countries like India where CD4 count and viral RNA load estimation cannot be routinely performed in large populations owing to its high cost. The HIV-related oral lesions are hence regarded as “sentinels and signposts” of HIV/AIDS and their early recognition and prompt management are of paramount importance in maintaining the health and prolonging the lives of patients with AIDS.

## Figures and Tables

**Table 1:** Revised CDC classification and case definition among adults (1993).

CD4-T Cell	Clinical categories		
	A Asymptomatic	B Symptomatic	C AIDS indicator
≥500/mm^3^	A_1_	B_1_	C_1_
200–499/mm^3^	A_2_	B_2_	C_2_
<200/mm^3^	A_3_	B_3_	C_3_
